# Biomarkers in Alzheimer’s disease

**DOI:** 10.1515/almed-2020-0090

**Published:** 2020-11-23

**Authors:** Manuel H. Janeiro, Carlos G. Ardanaz, Noemí Sola-Sevilla, Jinya Dong, María Cortés-Erice, Maite Solas, Elena Puerta, María J. Ramírez

**Affiliations:** Department of Pharmacology and Toxicology, School of Pharmacy and Nutrition, University of Navarra, Pamplona, Spain; IDISNA, Navarra’s Health Research Institute, Pamplona, Spain

**Keywords:** Alzheimer’s disease, β-amyloid, blood, cerebrospinal fluid, cognitive deficit, mild cognitive impairment, neuroimaging

## Abstract

**Background:**

Alzheimer’s disease (AD) is a progressive neurodegenerative disease. AD is the main cause of dementia worldwide and aging is the main risk factor for developing the illness. AD classical diagnostic criteria rely on clinical data. However, the development of a biological definition of AD using biomarkers that reflect the underling neuropathology is needed.

**Content:**

The aim of this review is to describe the main outcomes when measuring classical and novel biomarkers in biological fluids or neuroimaging.

**Summary:**

Nowadays, there are three classical biomarkers for the diagnosis of AD: Aβ42, t-Tau and p-Tau. The diagnostic use of cerebrospinal fluid biomarkers is limited due to invasive collection by lumbar puncture with potential side effects. Plasma/serum measurements are the gold standard in clinics, because they are minimally invasive and, in consequence, easily collected and processed. The two main proteins implicated in the pathological process, Aβ and Tau, can be visualized using neuroimaging techniques, such as positron emission tomography.

**Outlook:**

As it is currently accepted that AD starts decades before clinical symptoms could be diagnosed, the opportunity to detect biological alterations prior to clinical symptoms would allow early diagnosis or even perhaps change treatment possibilities.

## Introduction

Alzheimer’s disease (AD) is a progressive neurodegenerative disease. AD is the main cause of dementia worldwide and aging is the main risk factor for developing the illness. It is estimated that nowadays, 46.8 million people suffer from dementia worldwide, and the prevalence will increase due to population aging in the world, reaching by 2030 74.7 million people. Only four drugs are currently approved by the FDA to treat the illness: three cholinesterase inhibitors (donepezil, rivastigmine and galantamine) and an uncompetitive N-methyl-d-aspartate (NMDA) receptor modulator (memantine). Unfortunately, none of these available drugs slow or stop the progression of the disease. Therefore, AD is becoming one of the greatest sanitary challenges of this century.

**Figure 1:**
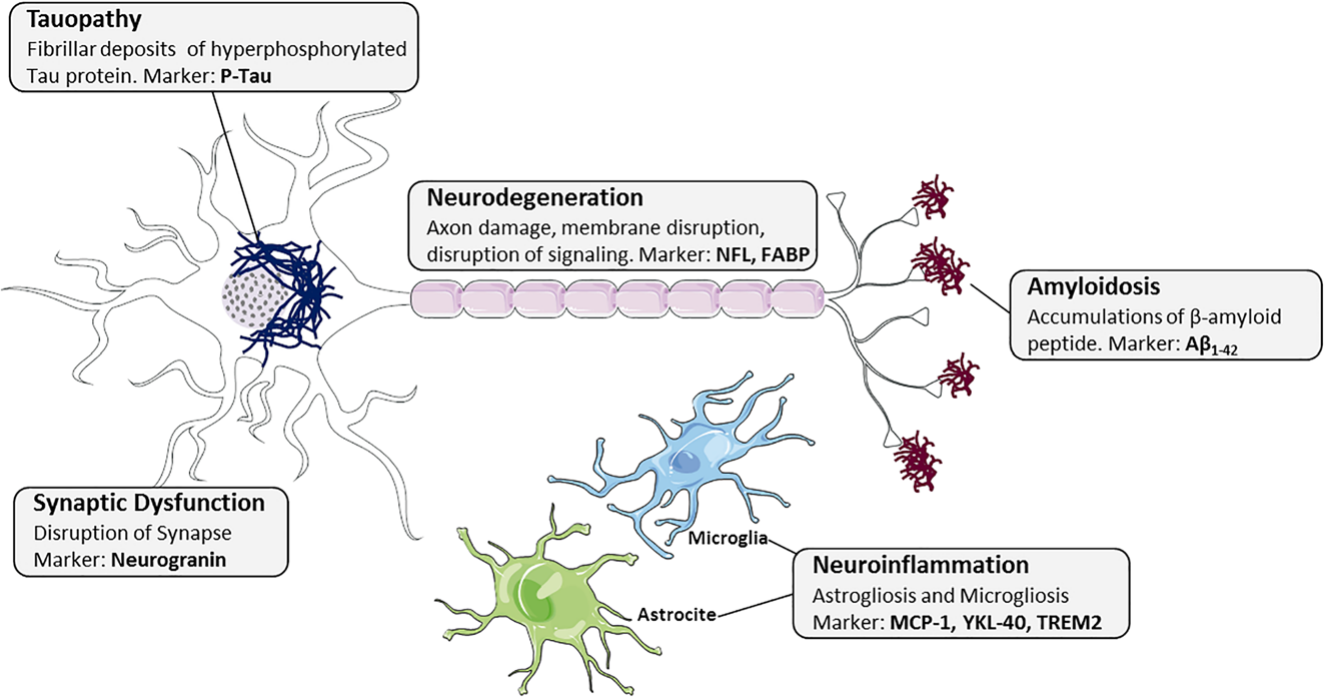
Biological markers of histopathological alterations in AD. Senile plaques, made up of accumulations of β-amyloid (Aβ) peptide, neurofibrillary tangles (NFT) formed by fibrillar deposits of hyperphosphorylated Tau protein (p-Tau), neuroinflammation, synaptic dysfunction and neurodegeneration.

Clinically, AD is defined by decline of memory and cognitive function. In addition, most patients suffer from neuropsychiatric symptoms called “behavioral and psychological symptoms of dementia”, such as depression, over-activity, psychosis or aggressive behavior. Histological features of AD are senile plaques, made up of accumulations of β-amyloid (Aβ) peptide, and neurofibrillary tangles (NFT), which are fibrillar deposits of hyperphosphorylated Tau protein (p-Tau). Other pathological events that seem to play a key role in the disease include synaptic dysfunction, inflammation or vascular dysregulation (Figure 1).

AD classical diagnostic criteria rely on clinical data. However, the development of a biological definition of AD using biomarkers that reflect the underling neuropathology is needed. In a complex syndrome, such as in AD, biomarkers would help in early diagnosis, staging of the disease, assessing prognosis and response to treatment. In addition, biomarkers could help to understand mechanisms of disease and develop new treatment strategies.

**Figure 2:**
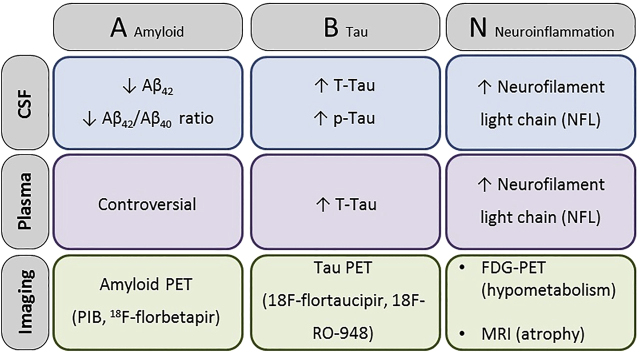
The AT(N) (amyloid-Tau-neurodegeneration) classification define “A” biomarkers as amyloid positron emission tomography (PET), cerebrospinal fluid (CSF) Aβ42 and CSF Aβ 42/40, “T” refers to Tau PET and CSF p-Tau, and “N” is shown by structural magnetic resonance imaging, fluorodeoxyglucose (FDG) PET, CSF total Tau (t-Tau) and neurofilament light chain protein (NFL).

According to the latest guidelines of the National Institute of Aging and Alzheimer Association (NIA-AA) [1], the term AD is applied whenever there is biomarker evidence of presence of Aβ plaques and NFT. Currently (Figure 2), the amyloid-Tau-neurodegeneration (AT(N)) classification define “A” biomarkers as amyloid positron emission tomography (PET), cerebrospinal fluid (CSF) Aβ42 and CSF Aβ42/Aβ40, “T” refers to Tau PET and CSF p-Tau, and “N” is shown by structural magnetic resonance imaging, fluorodeoxyglucose (FDG), PET, CSF total Tau (t-Tau) and neurofilament light chain protein (NFL) [2]. The aim of this review is to describe the main outcomes when measuring classical and novel biomarkers in biological fluids or neuroimaging.

## Biomarkers in fluids: cerebrospinal fluid

CSF reflects metabolic processes in the brain owing to direct contact between the brain and CSF and consequently, it has been become a useful fluid for AD diagnosis. CSF biomarkers could be considered more attractive than plasma biomarkers as they show better correlation with 11C-Pittsburgh compound B (PIB) PET imaging data and present more predictive values [3]. They also improve the certainty of diagnosis, mainly in prodromal phase or atypical presentations [4]. Nowadays, there are three classical CSF biomarkers for the diagnosis of AD: Aβ42, t-Tau and p-Tau.

### AD patients vs. healthy controls (HC) and other central nervous system (CNS) pathologies

Aβ peptides such us Aβ42, Aβ40, Aβ37, Aβ38, sAPPα and sAPPβ, were the first established molecular biomarkers for AD [5]. Among them, Aβ42 specially correlates with physiological and pathological symptoms with patients having lower CSF levels of Aβ42 compared to HC due to cortical amyloid depositions. However, plasma levels do not fulfill this association [3]. On the other hand, low levels of Aβ40 have also been shown to be related with AD. Nevertheless, its little size effect limits its use in diagnosis for AD. Although the differences are significant between AD patients and control cohorts, they are minimal. Moreover, Aβ37 and Aβ38 could help to distinguish AD from other close-related forms of dementia [6] but they are not really useful for clinical diagnosis alone as they show no significant differences between AD and controls in several studies [3].

sAPPα and sAPPβ are cleavage products of amyloid precursor protein (APP). sAPPα levels seems to be lower in atypical Parkinsonian syndrome compared with AD or Parkinson’s disease (PD), suggesting an alteration of APP metabolism [7]. However, sAPPα and sAPPβ are not really useful in clinical diagnosis alone, as the differences between AD patients and HC are not significant but especially sAPPα when taken together with other CSF biomarkers could help increase diagnosis accuracy [3]. t-Tau and p-Tau (Thr 181) were the next CSF biomarkers accepted. These markers are related to memory complaints and are present in higher levels in the CSF of AD patients. Mechanistically, t-Tau could be elevated according to cortical neuronal loss and p-Tau elevation would reflect cortical tangle formation. It is to note that p-Tau is characteristic of AD and helps to distinguish AD from other forms of dementia [3].

When these three markers (Aβ42, t-Tau and p-Tau) are measured together, they show better specificity and sensitivity than when measured alone [5]. Alterations in these markers, although to a lesser extent, are also present in mild cognitive impairment (MCI) compared to stable mild cognitive impairment subjects [5]. They are also able to predict the conversion of MCI to AD. The main problem when using these biomarkers is that, due to the inter-laboratory variability, there is a lack of consensus regarding limits to consider “low Aβ42 levels” and “high t-Tau levels” [3].

In addition to these classical biomarkers, new biomarkers are emerging in the literature [5]. Growing interest is developing toward neurodegeneration related markers, such as NFL. NFL is the light protein of neurofilament and, with either the medium or the heavy counterpart, makes up neurofilament bundles that determine axonal caliber and conduction velocity [8]. It is abundantly expressed in myelinated axons and is promptly released into the CSF and blood under axonal distress and degeneration. High level of NFL may reflect ongoing damage and demyelination [9]. NFL levels in several studies present significance and a big size effect indicating that axonal destruction is prominent in AD [5]. These high levels correlate with greater disease severity but not with disease duration. Moreover, NFL may contribute to increase diagnosis accuracy as it helps differentiating dementia with Lewy bodies (DLB) and AD from PD dementia as in DLB and AD there is an increase of NFL associated [7]. Increased age has been associated with increased NFL levels too [10].

YKL-40, a marker related to glial and astrocytic activation, and other proteins like carnosinase I, chromogranin A and neural cell adhesion molecule (NCAM), could be helpful in early diagnosis [3] but the effect size is moderate [5]. By contrast, monocyte chemoattractant protein-1 (MCP-1) or albumin ratio have not proven to be useful as diagnostic markers [5].

There are also some studies that show that α-synuclein is slightly higher in AD patients [11]. This hypothesis is contrary to the fact that CSF α-synuclein levels are lower in PD patients when comparing to controls. It should be noted that many of the studies suggesting this rise in CSF α-synuclein levels in AD are studies where patients were diagnosed according to clinical criteria alone so it is not possible to know if there was present a comorbid Lewy bodies pathology.

Finally, there are other new promising CSF biomarkers such as neurogranin or TREM-2. In the last five years, a few studies have appeared showing that the neuron-specific postsynaptic protein neurogranin (which is mainly expressed in regions affected in AD like cortex, hippocampus and amygdala) is higher in AD patients and this increase seems specific of AD as it is not seen in other neurodegenerative diseases. This increase may reflect synapse degeneration [12]. On the other hand, recent studies show that TREM2 which is an innate immune receptor expressed on the surface of microglia and is proteolytically processed and released as a soluble fragment (sTREM2) is increased in AD. Moreover, increased CSF sTREM2 levels have been associated with higher CSF t-Tau and p-Tau (Thr-181) [13]. Although their only a few studies published, the literature suggests that levels may increase early and peak prior to dementia.

To sum up (Table 1), the main CSF biomarkers to date that have shown more consistency and should be used in clinical practice and research are t-Tau, p-Tau, Aβ42 and these markers have reached 85–90% accuracy. In addition to these classical markers, and according to literature, it is suggested that NFL could be included as a new biomarker. Moreover, neurogranin seems a very promising and specific CSF biomarker with a large effect size but more studies are needed to check accuracy and usefulness.

**Table 1:** Biomarkers in AD.

Biomarkers	Description	CSF	Plasma	Usefulness in diagnostic
Aβ42	Marker of APP metabolism.	↓ Levels in AD patients. Great effect size.	No differences	Recommended for CSF diagnosis.
Aβ40	Marker of APP metabolism.	↓ Levels in AD patients. Small effect size.	No differences	Not really useful alone.
Aβ38	Marker of APP metabolism.	No differences between groups.		Not really useful alone. May help to distinguish AD from other close-related forms of dementia.
sAPPα	Cleavage product of APP.	No differences between groups.		Not really useful alone.
sAPPβ	Cleavage product of APP.	No differences between groups.		Not really useful alone.
t-Tau and p-Tau (Thr 181)	Markers related to memory complaints.	↑ Levels in AD patients. Large effect size.	↑ t-Tau levels in AD patients. Large effect size	P-tau is characteristic of AD. Recommended for CSF diagnosis.
NFL	Marker related to neurodegeneration.	↑ Levels in AD patients. Large effect size.		Recommended for CSF diagnosis.
NSE	Marker related to neurodegeneration.	↑ Levels in AD patients. Moderate effect size.	No differences	Could be useful for CSF diagnosis.
VLP-1	Marker related to neurodegeneration.	↑ Levels in AD patients. Moderate effect size.		Could be useful for CSF diagnosis.
HFABP	Marker related to neurodegeneration.	↑ Levels in AD patients. Moderate effect size.	No differences	Could be useful for CSF diagnosis.
Albumin-ratio	Marker for BBB function.	↑ Levels in AD patients. Small effect size.		Not really useful alone.
YKL-40	Marker for glial activation.	↑ Levels in AD patients. Moderate effect size.	↑ Levels in AD patients. Large effect but not significant.	Could be useful for CSF diagnosis.
MCP-1	Marker for glial activation.	↑ Levels in AD patients. Small effect size.	No differences	Not really useful alone.
GFAP	Marker for glial activation.	No differences between groups.		Not really useful alone.
Neurogranin	Marker for synapse degeneration.	↑ Levels in AD patients. Large effect size.		Specific for AD. Very promising but few studies published.
sTREM2	Marker related to neurodegeneration.	↑ Levels in AD patients. Moderate effect size.		Could be useful for CSF diagnosis but few studies published.
α-Synuclein	Presynaptic neuronal protein.	↑ Levels in AD patients. Negligible effect size.		Not really useful alone. Most of studies are performed with probable AD patients.

↑, increase; ↓, decrease. Data obtained from Janelidze et al. [14], Majbour [11], Olsson et al. [5], Suárez-Calvet [13] and Wellington et al. [12].

### AD patients vs. MCI patients

When comparing AD patients vs. MCI patients (Table 2), the useful of the biomarkers above-mentioned vary. Only t-Tau and p-Tau which keep being higher in AD patients, maintain a large effect size. On the other hand, Aβ42 levels remain lower in AD patients but the effect size is smaller. However, Aβ40 that presented a small decrease in CSF levels when comparing to HC, shows no differences comparing to MCI patients and Aβ38 that showed no differences when comparing AD patients vs. HC shows a negligible increase when comparing to MCI patients [5].

**Table 2:** Biomarkers in MCI-AD and MCI-Stable patients.

Biomarkers	CSF	Plasma	Usefulness in diagnostic
Aβ42	↓ Levels in AD patients. Smaller effect size than between patients with AD and controls.	No differences.	Recommended use.
Aβ40	No differences.	↑ Levels in AD patients. Negligible size effect.	Not really useful.
Aβ38	↑ Levels in AD patients. Negligible effect size.		Only two studies published. Small effect size.
sAPPα	No differences.		Not really useful.
sAPPβ	No differences.		Not really useful.
t-Tau and p-Tau (Thr 181)	↑ t-Tau and p-Tau levels in AD patients. Large effect size.		Recommended use.
Neurogranin	↑ Levels in AD patients. Moderate effect size.		Promising relative new biomarker. Few studies published.
YKL-40	↑ Levels in AD patients. Moderate effect size.		Promising relative new biomarker. Few studies published.

↑, increase; ↓, decrease; MCI, mild cognitive impairment. Data obtained from Janelidze et al. [14] and Olsson et al. [5].

Finally, neurogranin and YKL-40 levels were also higher with a moderate effect size in AD patients when comparing to MCI [14] but we could only find four studies to date.

In conclusion, when comparing AD patients and MCI patients, the best biomarkers are p-Tau and t-Tau, but neurogranin and YKL-40 could also be really helpful.

## Biomarkers in fluids: blood

The diagnostic use of CSF biomarkers is limited due to invasive collection by lumbar puncture with potential side effects. In this context, efforts are directed to discover reliable blood biomarkers. Plasma/serum measurements are the gold standard in clinics, because they are minimally invasive and, in consequence, easily collected and processed [15]. Moreover, patients can be followed up and screened over several years. However, developing blood biomarkers for AD has proven difficult and more challenging than CSF for several reasons. Firstly, changes are very small and heterogeneous because plasma/serum data reflect a broad spectrum of changes; not all necessarily related to AD. Secondly, only a fraction of brain proteins gets into the bloodstream and these have to be measured in a matrix containing high levels of plasma proteins, such as albumin and IgG, introducing an important risk of interference in analytical methods [16]. Third, in addition to dilution, brain proteins released into blood may be degraded by proteases, metabolized in the liver or cleared by the kidneys, which will introduce a variance that is unrelated to brain changes and is difficult to control [17]. Additionally, with the exception of certain blood biomarkers, like plasma Aβ [18] and plasma Tau [19], few studies have directly explored the relationship between CSF proteins and their blood-based analogs in the same cohort; therefore, the extent to which peripheral molecular changes accurately reflect CNS dynamics has yet to be characterized at large scale [20].

### Aβ levels

While numerous papers have described a high concordance with amyloid PET measures of plaque burden and a marked decrease in Aβ42 in CSF of AD patients, studies on plasma Aβ42 as a biomarker have been disappointing, with contradictory results. In this sense, different studies have shown that plasma Aβ42 and Aβ40 levels can be increased, reduced or even unchanged when AD and control individuals are compared [21], [ 22]. In addition, although previous longitudinal studies had shown that high plasma Aβ42 levels are a risk factor for developing AD [23], others have associated low plasma Aβ42/Aβ40 ratios with increased imminent risk for MCI and AD [24]. Thus, there is a general agreement that this factor is nor sensitive neither specific for early diagnosis [5].

Moreover, there seems to be no correlation between CSF and plasma Aβ levels [25], supporting the hypothesis that plasma Aβ levels reflect peripheral Aβ generation from other tissues more than AD brain pathology. Indeed, Aβ levels in blood fluctuate over time and among individuals [15]. Besides, it binding to other proteins and thus becoming trapped [26], plasma Aβ expression is influenced by medications [27] and, noteworthy, blood platelets contain high amounts of Aβ, which directly affects Aβ plasma levels [28].

In addition, the poor disease association might also be related to analytical shortcomings using ELISA methods or other standard immunoassays [29]. Some studies have described that, due to its hydrophobicity, Aβ peptides interact with many proteins of the plasma matrix such as albumin, α2-macroglobulin or lipoproteins among others [26]. This could cause epitope masking, hindering the recognition of up to 50% of these amyloid peptides in the immunoassays [30]. Therefore, this matrix effect could affect the reliability of Aβ peptide quantifications in an individual. In this context, Zetterberg’s group developed in 2011, a novel method based on the single-molecule array (Simoa) technique for measurement of Aβ42 in plasma [31]. This technique is based on immunocapture of the protein biomarker on magnetic beads, followed by the addition of enzyme-labeled detection antibody, and allows the exact quantification of Aβ42 with high sensitivity reducing matrix interferences. The Swedish BioFINDER cohort study tested this assay and found significantly lower plasma Aβ42/Aβ40 ratio in both MCI and AD cases as compared with controls [32]. Further, the same authors also developed an immunoprecipitation (IP) mass spectrometry (MS) selected reaction monitoring method for quantification of Aβ42 and Aβ40 in plasma. By using this technique, in a small pilot clinical study based on clinically diagnosed cases, only a trend for a reduction on both plasma Aβ42 and the Aβ42/Aβ40 ratio was observed in AD [33]. Interestingly, using a similar IPMS method, significantly lower Aβ42 concentration and Aβ42/Aβ40 ratio were found in amyloid PETpositive compared with PET negative cases [34].

Additional MS-based studies suggest that a ratio of a certain APP fragment (APP669-711) to Aβ42 or Aβ40/Aβ42 in plasma identifies Aβ-positive individuals with high sensitivity and specificity [35]. Specifically, plasma APP669-711/Aβ42 and Aβ40/Aβ42 ratios were higher in Aβ-positive than in Aβ-negative individuals [35]. These promising results encourage for further studies in larger clinical cohorts directed to evaluate plasma Aβ as a screening tool for brain amyloidosis and AD.

### Tau protein

Considering all plasma and serum biomarkers, t-Tau is the only that discriminates patients with AD from controls in most of the studies performed [5] showing a minor increase in plasma Tau in AD patients, although with too large overlap with controls to be diagnostically useful [19]. Interestingly, longitudinal data have showed significant correlations between plasma tau levels and future cognitive decline, as well as increases in atrophy measured by MRI and in hypometabolism measured by FDG-PET during the follow-up [19]. Noteworthy, tau protein in CSF has been found to be present as truncated fragments [36], thus it is possible that the development of assays based on specific antibodies for these Tau fragments will improve performance. Alternatively, measurement of t-Tau or p-Tau in neuron-enriched exosome preparations may improve performance for Tau as a blood biomarker [37], but further studies are needed to validate this finding.

### NFL protein

Nowadays, it is the most replicated blood biomarker for AD. The first Simoa method for quantification of the axonal NFL protein in blood samples was published in 2016 [38]. Indeed, many studies have shown higher NFL concentrations in patients with AD compared with age-matched control subjects [39] and other studies have described that blood NFL quantification could be used as a biomarker of neurodegeneration in the preclinical stage of AD [40].

Interestingly, while the change in the MCI group has been shown to be less pronounced, plasma NFL was highest in MCI cases with positive amyloid PET scans, and predicted faster cognitive decline, higher rate of future both brain atrophy (measured by MRI) and hypometabolism as measured by FDG-PET [39]. Furthermore, in familial AD studies, NFL appears to be altered around one decade before symptom onset [41] with levels correlating with expected estimated year of symptom onset as well as both cognitive and MRI measures of disease stage [40].

Nevertheless, it is important to note that NFL is not a specific feature for AD. Indeed, increased levels are found in many other neurodegenerative disorders, such as frontotemporal dementia, progressive supranuclear palsy, corticobasal syndrome, inflammatory conditions or acute traumatic brain injury [42]. Therefore, although the diagnostic specificity of NFL is lacking, the semiautomated measurement of NFL in blood offers the possibility of multiple sample collections to monitor disease progression and potentially treatment response. Another possible future application for plasma NFL is as a simple, noninvasive and cheap screening test, at the first clinical evaluation of patients with cognitive disturbances, primarily to rule out neurodegeneration.

## Image biomarkers: PiB PET, FDG PET and MRI

Imaging technologies have enabled a much deeper comprehension of the complexly inter-related pathophysiological mechanisms underlying AD. The two main proteins implicated in the pathological process, Aβ and Tau, can be visualized using PET. Furthermore, the local impact of neurodegeneration can also be minutely characterized: defects in brain glucose metabolism, regional tissue atrophy and brain network disruption can be assessed with PET, structural magnetic resonance imaging (MRI), and functional MRI, respectively. Thus, carefully extracting data from imaging the spatiotemporal dynamics of the pathophysiology of AD should offer further insights on the biology and assessment of the evolution of the disease in concrete patients.

### Amyloid-PET

Based on radiopharmaceuticals having a marked specificity and sensitivity for binding to amyloid plaques, such as PIB or 18F-florbetapir, this technique permits the visualization of aggregated forms of Aβ (being unable to detect the more pathogenic oligomeric soluble forms). Notably, there is a broad consensus that a marked increase in Aβ accumulation occurs in the AD brain. Mapping brain Aβ has revealed that large areas in the medial and lateral association cortex are especially prone to Aβ deposition even in individuals without dementia [43]. Importantly, the first stage in the development of AD seems to occur with Aβ deposition in the medial parietal cortex. Indeed, in patients suffering from autosomal-dominant Alzheimer’s disease (ADAD), it seems that Aβ deposition takes place in the striatum and medial temporal lobe (MTL) up to 20 years before disease onset [44], and in the case of AD on a background of aging, Aβ deposition begins in the medial parietal and frontal cortex [43]. Importantly, Aβ accumulation takes place throughout the association cortex, but correlates very poorly with symptoms [45]. It is worthy to note that amyloid-PET might be the only neuroimaging technique in which more or less robust cut-points for AD are flourishing: a profoundly exhaustive study comparing diverse cut-point-establishing methods has pointed that an amyloid-PET universal centiloid value of 19 seems to be a well determined cut-point in AD [46].

### Tau-PET

The relative newness of Tau-PET ligands implies that not much longitudinal studies regarding Tau accumulation have been performed. However, following a first generation of Tau tracers (e.g. 18F-flortaucipir) whose major problem was off-target binding, a second generation was developed (e.g. 18F-RO-948) and currently some studies are shedding further light on the spatiotemporal dynamics of Tau accumulation. Although the timing and physical location of Tau pathology are uncertain, it might develop from the MTL: significantly increased Tau tracer retention levels have been found in various regions of patients suffering from AD, including the MTL, inferior lateral temporal, posterior cingulate and lateral parietal regions [47], [ 48]. The use of second-generation Tau tracers has allowed to confirm that AD patients exhibit a higher tracer signal in medial temporal areas as well as throughout the posterior cingulate, lateral parietal and occipital lobes and prefrontal cortex [49]. However, both experimental and clinical validation of Tau tracers are still very scarce [50], and the amount of hyperphosphorylated Tau present in the brain below the detectable threshold is not known yet. These are some of the reasons why a reliable cut-point for Tau-PET has not been produced for the diagnosis of AD [46].

### FDG-PET

Taking advantage of the glucose analog FDG, the use of PET allows mapping brain glucose metabolism. Thus, it permits to characterize metabolic activity, whose deficits are normally interpreted as a loss of neuronal and/or synaptic function, but could also reflect alterations in glial cell function, increasingly considered nuclear to understand AD neuropathology. Importantly, the spatial location of hypometabolism differs among the different AD syndromes, conceding an opportunity to discriminate the actual phenotype: it begins in the MTL years before the onset of the disease and then spreads to the lateral parietal cortex in ADAD [51]; it is strongly correlated with Tau deposition in early-onset Alzheimer’s disease [52]; and it predominates in the MTL and medial parietal lobe in late-onset Alzheimer’s disease [53]. On the contrary, hypometabolism does not follow a distinctive pattern in normal aging, despite following some frontal predominance. Finally, as the disease develops, a characteristic topographical pattern of brain hypometabolism takes place in the temporoparietal and posterior cingulate cortices [54]. However, the grade and location of hypometabolism needed to establish a trustworthy threshold value for AD patients has not been elucidated yet.

### Structural MRI

Depending on a signal that originates from within the body, structural MRI permits accurate, noninvasive measurement of brain tissue volumes, thus being able to properly locate pathophysiological features such as regional brain atrophy, gray matter loss or white matter damage. The main findings achieved by brain volumetric measurement techniques have identified gray matter atrophy occurring of MTL structures in AD, but also in MCI, seeming that the most consistent biomarker for conversion from MCI to AD is the atrophy of the left MTL [55]. Further parameters predicting the conversion from MCI to AD include reduction in hippocampal and parahippocampal gray matter volumes and the presence of a more vague cortical loss in AD [56]. Furthermore, diffusion imaging techniques, which take advantage of the fact that microscopic water diffusion is anisotropic in the human brain and use it to identify white matter tracts, have detected deterioration of some major tracts in AD. Mainly, a disruption of the cingulum (which connects MTL structures with the rest of the brain) occurs during the transitional stages from normal aging to AD. Damage to the uncinate fasciculus, involved in memory and emotional processes, has also been found [57]. Nonetheless, the number of brain cells or white matter tracts that must be lost to detect and define atrophy in AD is not currently known, and the amount of evidence required to produce a reliable threshold value of cortical thinning is currently lacked [46].

### Functional MRI

Measuring MRI signals that reflect tissue perfusion, functional MRI (fMRI) is based on the fact that different brain regions that exhibit synchronous signal fluctuations belong to the same brain network. A disruption of these signals is interpreted as an alteration in network connectivity and thus allows studying changes in brain connectivity. Remarkably, a loss of functional connectivity affecting critical regions of the default-mode network (DMN), such as the posterior cingular cortex, has been described to precede gray matter loss in AD [58]. Given that other dementias are constituted by alterations of different networks [59], disruption of the DMN connectivity signals measured by fMRI could possibly become a biomarker pointing to AD. Last but not least, disconnection occurring in the hippocampus and medial prefrontal cortex also seem to portray the characteristic AD neuropathology [60]. As some of the other imaging techniques described, the lack of evidence has prevented the establishment of a cut-point for fMRI signal alterations to diagnose or discriminate AD patients.

## Future perspectives: novel biomarkers and fluids

Active research is being developed in the search for noninvasiveness and easy access biological setups, such as saliva [61]. Up-to-date, conflicting and nonconclusive results have been obtained when measuring Aβ42 [62], [63], [64] or p-Tau [64], [ 65] in this fluid [66]. It is to note that circadian variation could notably influence the composition of saliva. Furthermore, the oral health or medication could affect biomarkers detection. Therefore, is necessary to standardize work protocol to obtain a reproducible result [67].

In the last years, miRNAs in blood have appeared as promising early AD biomarkers. MiRNAs are short noncoding molecules that work as epigenetic factors, regulating gene expression post-transcriptionally, by binding to complementary sequences on target mRNAs. MiRNAs are often encapsulated in exosomes, microvesicles or apoptotic bodies, structures that can transport other substances. Exosomes containing miRNAs can cross the blood-brain barrier and mediate the cross-talk between blood, brain and CSF [68]. MiRNAs carry out physiological but also pathological function, and it seems that some of them are dysregulated in AD. The most studied in AD have binding sites in mRNAs that code for proteins that play a key role in the disease: APP, Tau, BACE, PSEN2, MAPK … Under physiological conditions, exosomes are capable of transporting accumulated proteins (Aβ and Tau) to lysosomes or extracellular plasma for degradation, but under pathological conditions, this clearance is disrupted. However, even though no conclusive results have been described, it is to mention a recent review describing that has-miR-146a, has-miR-125b and hsa-miR135a could be differentially express in blood and CSF in AD compared with control, other neurological diseases or even with MCI individuals [68].

## Conclusions

While AD classical diagnostic criteria rely on clinical data, newer criteria are needed to identify the disease in its earlier stages. It is currently accepted that AD starts decades before clinical symptoms could be diagnosed. The opportunity to detect biological alterations prior to clinical symptoms would allow early diagnosis or even perhaps change treatment possibilities.
